# Transmission electron microscopic observations of nanobubbles and their capture of impurities in wastewater

**DOI:** 10.1186/1556-276X-6-295

**Published:** 2011-04-05

**Authors:** Tsutomu Uchida, Seiichi Oshita, Masayuki Ohmori, Takuo Tsuno, Koichi Soejima, Satoshi Shinozaki, Yasuhisa Take, Koichi Mitsuda

**Affiliations:** 1 Division of Applied Physics, Faculty of Engineering, Hokkaido University, Sapporo 060-8628, Japan; 2 Department of Biological and Environmental Engineering, Graduate School of Agricultural and Life Sciences, The University of Tokyo, Tokyo 113-8657, Japan; 3 Department of Biological Science, Faculty of Science and Engineering, Chuo University, Tokyo 112-8551, Japan; 4 Tsuno Rice Fine Chemicals Co., Ltd., Wakayama 649-7194, Japan; 5 R&D Center, Mayekawa MFG. Co., Ltd., Ibaraki 302-0118, Japan; 6 Mixing Project, Nikuni Co., Ltd., Kanagawa 213-0032, Japan

## Abstract

Unique properties of micro- and nanobubbles (MNBs), such as a high adsorption of impurities on their surface, are difficult to verify because MNBs are too small to observe directly. We thus used a transmission electron microscope (TEM) with the freeze-fractured replica method to observe oxygen (O_2_) MNBs in solutions. MNBs in pure water and in 1% NaCl solutions were spherical or oval. Their size distribution estimated from TEM images close to that of the original solution is measured by light-scattered methods. When we applied this technique to the observation of O_2 _MNBs formed in the wastewater of a sewage plant, we found the characteristic features of spherical MNBs that adsorbed surrounding impurity particles on their surface.

**PACS**: 68.03.-g, 81.07.-b, 92.40.qc

## Introduction

Small bubbles, such as microbubbles (MBs; usually range from 10^-4 ^to 10^-6 ^m in diameter) and nanobubbles (NBs; less than 10^-6 ^m in diameter), have various properties that differ from macroscopic bubbles (greater than 10^-3 ^m in diameter). For example, smaller bubbles have lower buoyancies, so they take longer to reach the liquid surface and thus they have longer residence times. Also micro- and nanobubbles (MNBs) have either negative or positive zeta potentials [1,2]. This property inhibits the easy agglomeration or coalescence of bubbles and results in the relatively uniform size distribution of MNBs. Additionally, the smaller the bubble, the larger the specific interfacial area. Thus, the efficient physical adsorption of impurities included in the solutions on the bubble surface is expected. MNBs have now attracted attention for applications in engineering areas such as the sewage treatment of wastewater by air flotation [3,4,5,^6^] detergent-free cleaning of adsorbed proteins [7,8].

Moreover, as expected from the Young-Laplace equation, the smaller the bubble, the higher the pressure inside it. Therefore, the driving force for mass transfer from gas phase to surrounding liquid increases with decreasing bubble size. The gas solubility and the chemical reactions at the gas-liquid boundary are thought to be enhanced injecting the MNBs instead of normal aeration of macroscopic bubbles. MNBs have thus also attracted much attention as a functional material in the biological area, such as accelerating metabolism in vegetables [9], aerobic cultivation of yeast [10], and sterilization by a mixture of ozone MBs [11].

MBs have been observed by an optical microscope [12,13] to shrink in water with dissolving gas molecules in surrounding water and with increasing internal gas pressures. However, when bubbles become smaller than the spatial resolution of the optical microscope, it is difficult to recognize whether the bubble finally disappears by dissolving in water or it remains in water as a NB. The lifetime of MNB is also not agreed upon. Early studies suggested that the life time of NBs (10 to 100 nm in radius) in water was between 10^-6 ^and 10^-4 ^s (estimated by the simulation [14]), or that no evidence of carbon dioxide NB existence was found in ethanol solution by static and dynamic light scattering and infrared spectroscopy [15]. These conclusions are inconsistent with those observed in the engineering or biological investigations reported previously. In order to use MNBs for such practical applications, it is necessary to observe them directly and to reveal their fundamental properties.

The present study focused on finding evidence of existing MNBs and their functions, especially NBs, in the liquid phase using a transmission electron microscope (TEM) along with the freeze-fractured replica technique. This technique has usually been applied for biological investigations but is also useful for investigating the microstructures and the dynamic features of MNBs in solution when a small droplet is quenched at liquid nitrogen temperature [16,17,^18^]. To verify the effectiveness of this technique, we first observed oxygen (O_2_) MNBs formed in pure water. We then applied this technique to a commercially obtained MNB solution containing 1% NaCl, and finally to a wastewater solution from a sewage plant.

## Experimental

We prepared a pure MNB solution by introducing pure O_2 _gas (Nissan Tanaka Co., Saitama, Japan; purity of 99.999%) into the ultra-high purity water (Kanto Chem. Co., Inc., Tokyo, Japan) with a MNB generator (Aura Tec Co. Ltd., Fukuoka, Japan, OM4-MDG-045) operating for 120 min at 293 K. Since this sample preparation procedure was similar to that used in the previous work [19], the average bubble size was estimated as 140 nm, and the zeta potential of bubbles to be -40 mV. Based on dynamic light scattering (DLS) measurement (Quantum Design Japan Inc., Tokyo, Japan, Nanosight-LM10), the number concentration of MNBs was estimated to be on the order of 10^7 ^cm^-3 ^of solution under the same sample preparation conditions.

The details of the replica sample preparation were mentioned elsewhere [20], so we explain them just briefly here. A small amount of this solution (10 to 20 mm^3^) was put on an Au-coated Cu sample holder and was rapidly frozen by immersing it into a liquid nitrogen bath. In this condition, the freezing rate ranged from 10^2 ^to 10^3 ^K min^-1^. The frozen droplet was then fractured under vacuum (10^-4 ^to 10^-5 ^Pa) and low temperature (approximately 100 K) to reduce the formation of artifacts. The replica film of this fractured surface was prepared by evaporating platinum and carbon (JEOL Ltd., Tokyo, Japan, JFD-9010) prior to removing the replica film from the ice body by melting. We used a field-emission gun-type TEM (JEOL Ltd., Tokyo, Japan, JEM-2010) to observe the replica film at a 200-kV acceleration voltage. An imaging plate (Fujifilm Co., Tokyo, Japan, FDL-UR-V) was used for acquiring the observed image.

The same processes were used for MNBs in the dilute salt solution to investigate the effect of solutes on MNB existence in solutions. The O_2 _MNBs in water containing 1% NaCl were donated by REO Research Institute (Miyagi, Japan). We prepared the replica sample for this solution just after its delivery, when it took more than one week after the MNB formation.

Based on the above fundamental investigations for observing MNBs in solutions by the present experimental method, we observed the features of MNBs in the polluted water that was actually used for an engineering application. The polluted solution was sampled from a sewage plant as the wastewater of inositol extraction from defatted rice bran at Tsuno Rice Fine Chemicals Co., Ltd. (Wakayama, Japan). The polluted solution was expected to include several water-soluble impurities, such as glucide derived from rice starch (approximately 2 wt%) and calcium sulfate (almost saturated at room temperature), as well as insoluble micro particles. The original wastewater sample was milky-white with no macroscopic impurities. In this prototype plant manufactured by Mayekawa MFG. Co., Ltd., Ibaraki, Japan, pure O_2 _gas was aerated through the MNB generator (Nikuni Co., Ltd., Kanagawa, Japan, MBG20ND04Z-1GB) for 5 min. After aeration, some amounts of macroscopic insoluble impurities were observed in the bulk wastewater, which could have come from the grime in the plant system. However, the volume of sampled solutions used for the replica preparation was so small that we could exclude such macroscopic impurities easily. Solution droplets for the replica preparation were quenched just after the 5-min aeration at the plant site. The replica of the quenched sample was then prepared in the laboratory after transportation while maintaining the cryogenic temperature.

## Results and discussion

**Figure 1:**
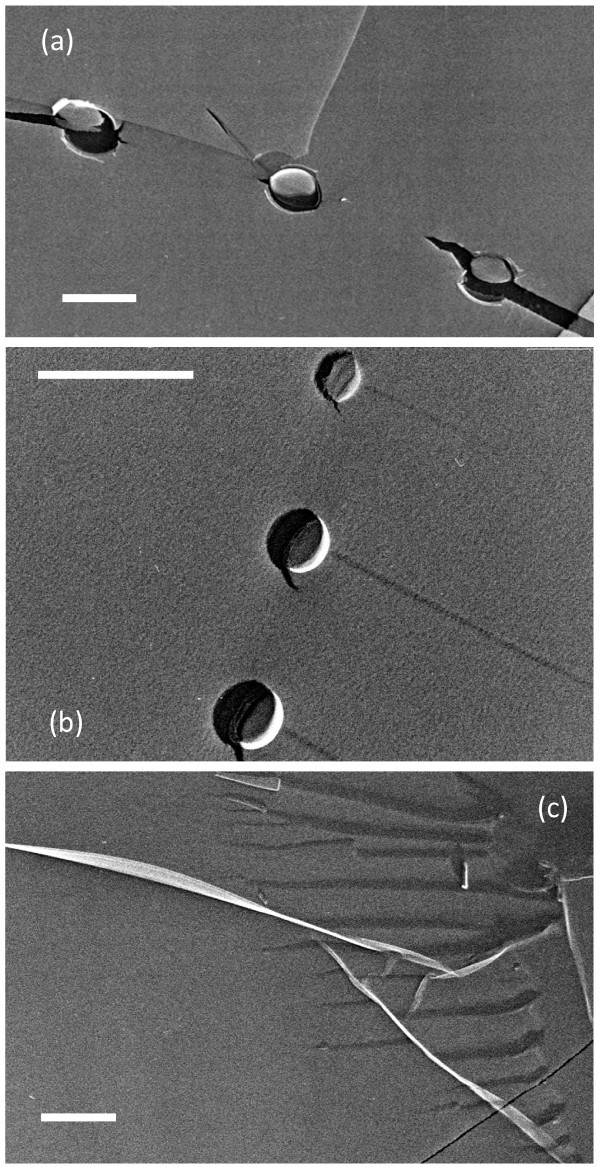
**Various TEM images of freeze-fractured replica of pure O_2 _MNBs in pure water**. Spherical or oval NBs of **(a) **500 nm in diameter or **(b) **200 nm in diameter were located in ice crystallites (smooth surface) or on their grain boundaries. **(c) **The replica sample of pure water without aeration was shown as a control. Each scale bar indicates 500 nm.

**Figure 2:**
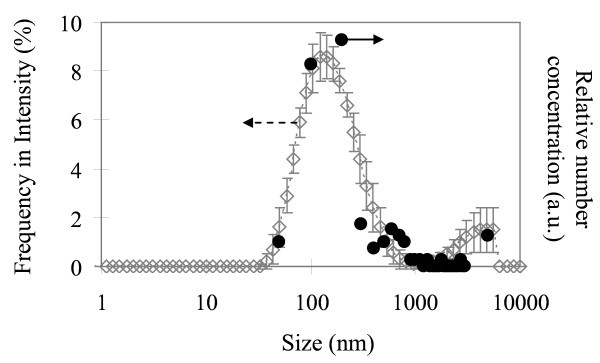
**Comparison of size distributions of O_2 _MNBs formed in pure water**. The size distribution of MNBs obtained from TEM images of replica samples prepared just after aeration (solid circles with arbitrary unit, *n *= 114) is similar to that measured by a dynamic light scattering method (open diamonds with error bars and a smoothed line), which was reproduced from Ushikubo et al. [19].

TEM images indicated that most of the observed areas on the replica samples for the pure water including O_2 _MNBs were smooth, and that a small number of objects were observed. Based on the observation in an early study [20,21], the smooth area corresponded to the ice crystallite formed during quenching, and the objects were resulted from the textures formed during ice crystal growth or from the aggregation of a small amount of impurities included in the original solution. In addition, we found several spherical or oval holes in TEM images, which had relatively uniform sizes ranging from 10^-6 ^to 10^-7 ^m (Figure 1a, b). Since the number concentration of these holes was estimated to be 10^7 ^to 10^8 ^cm^-3^, which was obviously greater than that observed on the replica samples of pure water without aeration (as the control, see Figure 1c), most of these holes were considered to be MNBs that originally existed in solutions. This is supported by the facts that the number concentration of MNBs estimated from TEM images corresponded to the value expected from DLS measurements (10^7 ^cm^-3^), and that the size distributions of MNBs observed on the replica samples coincided qualitatively with those obtained in the original bulk MNB water [19] (Figure 2). The quantitative disagreement of the two distributions observed in this figure could be caused by that the size distribution from TEM images being slightly modified because the present observations were based on a limited amount of sample and observed TEM images were random but in small numbers (here *n *= 114). Therefore, we concluded that we could evaluate the existence of O_2 _MNBs formed in pure water by using our freeze-fractured replica method. This conclusion also supports the validity of the replica method for application to MNB studies as mentioned previously [16,17,^18^] and indicates that the lifetime of MNBs formed in pure water was long enough to prepare the samples with quenching.

**Figure 3:**
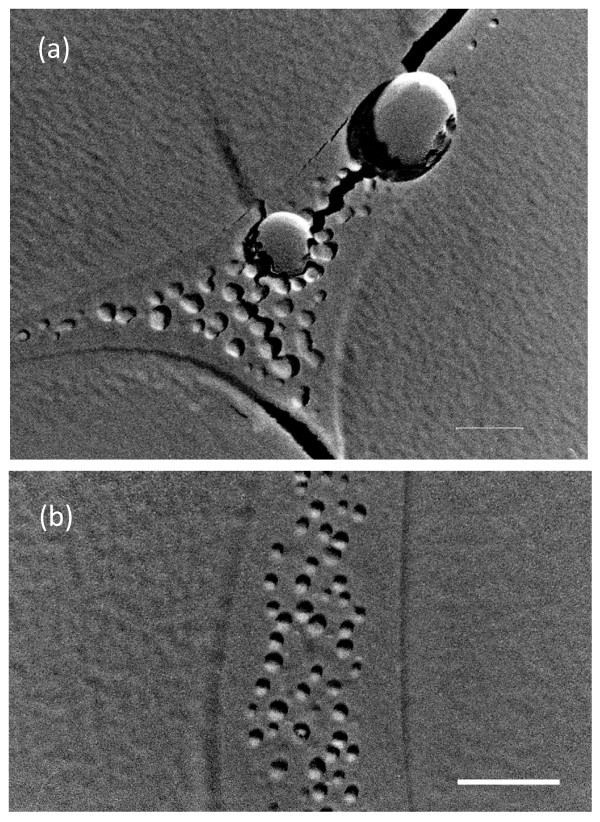
**TEM images of freeze-fractured replica of 1% NaCl solution containing O_2 _MNBs**. Scale bar indicates 200 nm. **(a) **Precipitated fine impurity particles (10 to 60 nm in diameter) and MNBs (200 and 300 nm in diameter) coexisted at the grain boundary of ice crystallites. Some fine particles were located around small MNBs but did not cover the entire bubble surface. **(b) **Replica sample of 1% NaCl solution without MNBs shown as a control.

In order to examine the interaction between MNBs and additives in the solution, we observed a dilute NaCl solution containing O_2 _MNBs. The obvious difference in TEM images of these samples from those in pure MNB water was that fine particles (less than 100 nm in diameter) were observed on the grain boundary of ice crystallites (Figure 3a). These fine particles were also observed in the control (no MNB sample, Figure 3b). MNBs were also simultaneously trapped on the grain boundary in this figure. Based on the analogous features of disaccharide solutions [20,21], the ice crystallites were formed during the sample quenching process, and the fine particles were the agglomeration of condensed salts dissolved in the original solution due to the freeze-condensation mechanism. The remaining area in the grain boundary is considered to be the glass state of the solution. The shape and size of MNBs in 1% NaCl solution seemed to be similar to those in pure water. Its number concentration was slightly lower than that in pure water system, which may have resulted from the sample being prepared more than 1 week after aeration. This result is qualitatively consistent with the DLS measurements in pure water [19]. The addition of a small amount of NaCl is expected to play a positive role of stabilizing MNBs in engineering applications. However, we could not find obvious characteristics in our TEM images as reported for the sample with surfactants [17]. Since there are conflicting claims for the effect of ionic solutions on MNB stabilities [22], further systematic investigations are required for understanding the effect of additives on the lifetime of MNBs.

**Figure 4:**
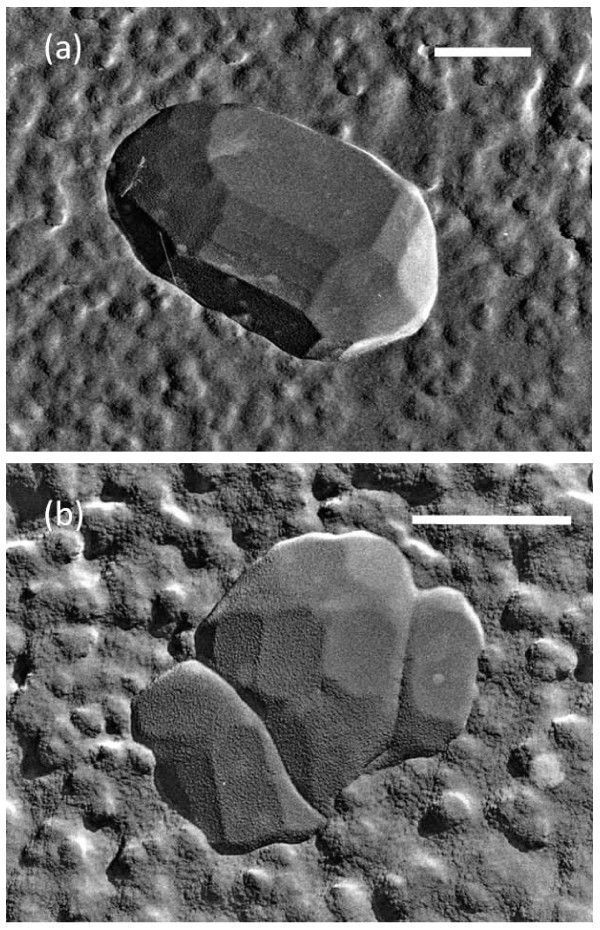
**Various TEM images of freeze-fractured replica of the wastewater containing MNBs**. Each scale bar indicates 500 nm. An ice crystallite with a faceted smooth surface was located in the center of each picture **(a, b)**, and surrounded by a rough surface composed of fine particles (impurities). The remaining area around the particles is the glass state of the solution.

The replica observations for the wastewater with MNBs exhibited obviously different images from those mentioned above. Several parts of the replica samples prepared from the wastewater had a rough surface including many fine particles (less than 10^-7 ^m in diameter) as depicted in Figure 4a, b. These fine particles resulted from either invisible small particles or from the agglomeration of the condensed soluble impurities such as glucide or calcium sulfate, both of which are considered to be included in the original wastewater. In addition, we sometimes found micron-sized ice crystallites among the fine particles, and found that they had crystalline facets with a smooth surface (center of Figures 4a, b). These ice crystallites are considered to be formed in the polluted solution during the sample quenching. The remaining area around the fine particles is the glassy body. The smooth surface of ice crystallite suggested that the observed rough surface surrounding the ice did not come from any artifacts on the replica during the sample preparation, such as frost deposit. The analogous features for disaccharide solutions [20] suggested that the original solution included a relatively high concentration of impurities because the crystallites were small and faceted, which indicated they grew slowly due to the impurities.

**Figure 5:**
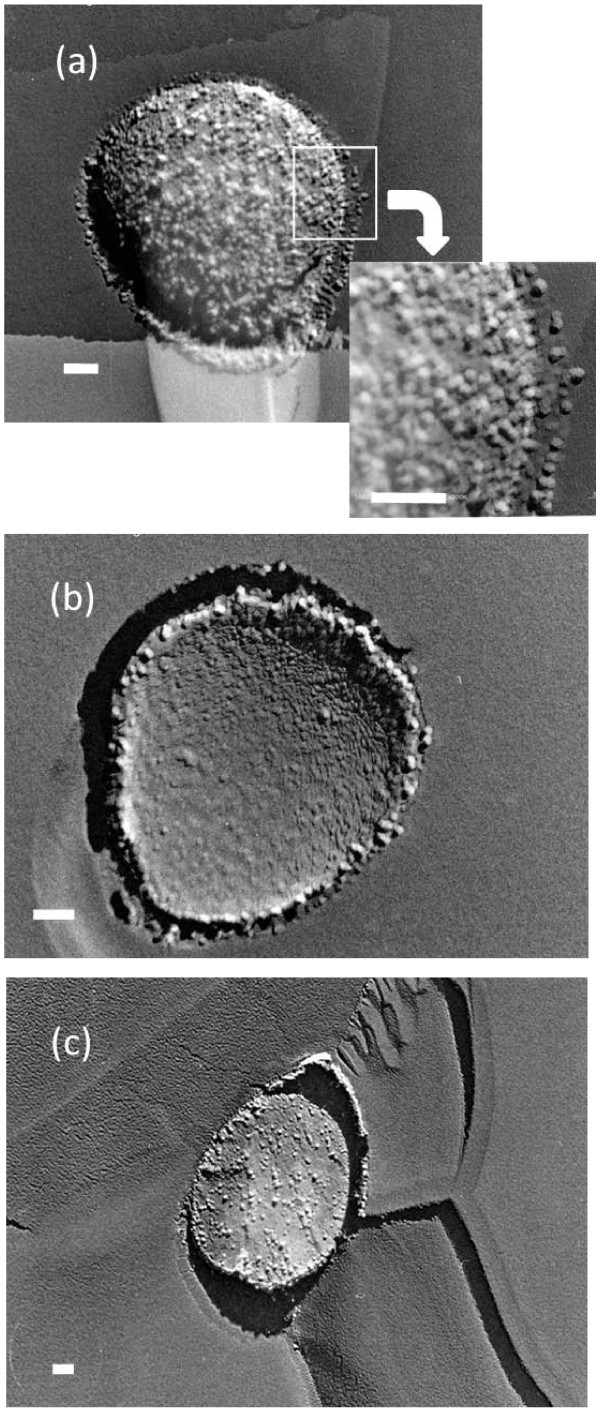
**Various TEM images of freeze-fractured replica of the wastewater containing O_2 _MNBs**. Each scale bar indicates 100 nm. **(a, b) **The MNB (850 nm in diameter) located in the center of each picture adsorbed many fine particles (20 nm in diameter) on its surface. The extended picture in **(a) **depicts the bubble-solution boundary indicating the process by which fine particles were attracted to the bubble surface. In contrast, no fine particles were observed around the MNB. **(c) **MNBs that captured fine particles were also located on the grain boundary between ice crystallites.

In contrast, several replica images in the same quenched sample exhibited a relatively wide smooth area similar to that of the pure water sample. In that area, we found some spherical objects that had adsorbed a large number of fine particles on their surface (Figure 5). These spherical objects ranged from 5 to 9 × 10^-7 ^m in diameter, which corresponded to the expected size of the MNBs formed in the solution. The fine particles on the spherical objects (or NBs) were 2 to 3 × 10^-8 ^m in diameter. Since no fine particles were observed around the NB, we postulated that these fine particles were impurities originally included in the wastewater and located around the MNB. Therefore, Figure 5 clearly indicates that MNBs in the wastewater trapped impurities existed around them on their surfaces and concentrated impurities during their residence time until quenching. This is the first direct observation of a typical property of MNBs, that is, MNBs adsorb effectively and concentrate impurities in solutions on their surface, which results in separating impurities from solutions.

**Figure 6:**
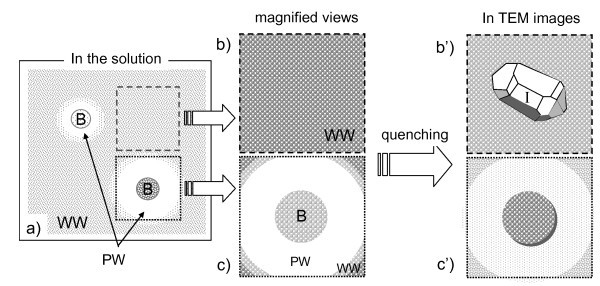
**Illustrations of adsorption properties of MNBs in wastewater and of their quenching features**. **(a) **The original wastewater (WW) includes both impurities (small dots) and several amounts of MNBs (*B*). Since a MNB sweeps impurities around it on the surface, the swept area is less polluted (white area around *B*) and the surface of the MNB is covered by impurities (small dots). When this solution is quenched and the replica samples are prepared on area **(b)**, no MNBs with homogeneously dispersed impurities were observed. We can observe the TEM image of **(b') **fine particles homogeneously dispersing with a small ice crystallite (*I*) formed in the quenching process (related to Figure 4). In contrast, when the replica sample was prepared on area **(c) **including the MNB surrounded by purified water (PW), the observed TEM image was **(c') **the MNB adsorbing fine particles on its surface in smooth ice crystallites (related to Figure 5).

Compared to the fine particles observed in 1% NaCl solutions (Figure 3), the fine particles in the wastewater adsorbed on a MNB homogeneously. This may indicate that the fine particles on MNBs in the wastewater were not the precipitation of soluble impurities but the insoluble small particles originally existing in the solution. The homogeneous distribution of fine particles near the MNB surface (within 50 nm from the interface, see the extended figure of Figure 5a) seemed to suggest that fine particles in the wastewater tended to be attracted to the MNB. Based on these TEM images of replica samples from the wastewater (Figures 4 and 5), the impurity adsorption of MNBs in the wastewater can be described as follows (Figure 6). If the wastewater including both fine particles and soluble impurities at a relatively high concentration were solely quenched at liquid nitrogen temperature, fine particles could be fixed homogeneously in the glass state of the solution, and some ice crystallites would be formed by the freeze-condensed mechanism (Figure 6b, b'). Since the impurity concentration was high, the ice crystallite nucleation was limited, and its growth was slow enough to form the crystalline facets. This result is related to the fact that the area of the glass state with fine particles exceeded that of the ice crystallites. However, if the solution included MNBs, the insoluble particles would be collected on the MNBs by the attractive force between them in solutions (Figure 6c). The mobility of MNBs was not so high and the attractive force would only be present at limited distances, so the sweep area of a MNB in the solution was limited to only around the bubble (Figure 6a). Figure 5 depicts the quenched features of this condition (Figure 6c'). Therefore, it is conceivable that the application of MNBs to the engineering aspects is effective, but its total effectiveness would directly depend on the number concentration of MNBs and on their residence time.

## Conclusions

We performed the TEM observation of the freeze-fracture replica to investigate the morphological features of MNBs in solutions. The MNBs in pure water were spherical or oval, and their size distribution ranged from 10^-6 ^to 10^-7 ^m, which was close to those obtained by the usual method for the MNB characterization (DLS measurement). Similar MNB features were observed in the TEM images of the 1% NaCl solution system, although the interaction between MNBs and the precipitated solute particles was not obvious. These results confirmed the feasibility of applying TEM observation with the freeze-fracture replica method for investigating MNBs in solutions.

When we applied this method to MNBs aerated in the wastewater of a sewage plant, we observed the special features of MNBs that collected surrounding impurities on their surfaces. The detailed investigation of obtained TEM images of the same wastewater suggested that the sweep area of a MNB in the solution was limited. Therefore, it is conceivable that the application of MNBs to engineering aspects is effective, but its total effectiveness would strongly depend on the number concentration of MNBs and on their lifetime.

## Abbreviations

MBs: microbubbles; MNBs: micro- and nanobubbles; NBs: nanobubbles; TEM: transmission electron microscope; DLS: dynamic light scattering.

## Competing interests

The authors declare that they have no competing interests.

## Authors' contributions

TU carried out TEM observations with sample preparations, and performed the entire observation analysis. TU, SO, and MO conceived of the study and participated in the experimental design and coordination. They also drafted the manuscript. SO prepared MNBs in pure water and analyzed the particle size distribution with DLS. TT, KS, SS, YT, and KM participated in the design and construction of the sewage plant and performed the sample preparation of MNBs in the wastewater. All authors read and approved the final manuscript.
